# The Prognostic Value of the DNA Repair Gene Signature in Head and Neck Squamous Cell Carcinoma

**DOI:** 10.3389/fonc.2021.710694

**Published:** 2021-07-30

**Authors:** Ruijie Ming, Enhao Wang, Jiahui Wei, Jinxiong Shen, Shimin Zong, Hongjun Xiao

**Affiliations:** Department of Otorhinolaryngology, Union Hospital, Tongji Medical College, Huazhong University of Science and Technology, Wuhan, China

**Keywords:** head and neck squamous cell carcinoma, DNA repair gene, prognostic signature, immune microenvironment, tumor mutation burden, drug sensitivity

## Abstract

**Purpose:**

To construct a prognostic signature composed of DNA repair genes to effectively predict the prognosis of patients with head and neck squamous cell carcinoma (HNSCC).

**Methods:**

After downloading the transcriptome and clinical data of HNSCC from the Cancer Genome Atlas (TCGA), 499 patients with HNSCC were equally divided into training and testing sets. In the training set, 13 DNA repair genes were screened using univariate proportional hazard (Cox) regression analysis and least absolute shrinkage and selection operator (LASSO) Cox regression analysis to construct a risk model, which was validated in the testing set.

**Results:**

In the training and testing sets, there were significant differences in the clinical outcomes of patients in the high- and low-risk groups showed by Kaplan-Meier survival curves (*P* < 0.001). Univariate and multivariate Cox regression analyses showed that the risk score had independent prognostic predictive ability (*P* < 0.001). At the same time, the immune cell infiltration, immune score, immune-related gene expression, and tumor mutation burden (TMB) of patients with HNSCC were also different between the high- and low-risk groups (*P* < 0.05). Finally, we screened several chemotherapeutics for HNSCC, which showed significant differences in drug sensitivity between the high- and low-risk groups (*P* < 0.05).

**Conclusion:**

This study constructed a 13-DNA-repair-gene signature for the prognosis of HNSCC, which could accurately and independently predict the clinical outcome of the patient. We then revealed the immune landscape, TMB, and sensitivity to chemotherapy drugs in different risk groups, which might be used to guide clinical treatment decisions.

## Introduction

Head and neck squamous cell carcinoma (HNSCC) is a type of tumor that originates from the squamous epithelium of the head and neck areas, including the mucous membranes of the lips, tongue, pharynx, larynx, and others (1). HNSCC is currently one of the most common malignant tumors worldwide, with morbidity and mortality accounting for 3.6 and 3.4% of all malignant tumors in 2020, respectively (2). HNSCC is highly malignant, and there are no specific prognostic-related biomarkers for clinical application. Therefore, prognostic-related biomarkers with clinical applicability are urgently required.

DNA damage and repair play important roles throughout the life of a cell (3). DNA damage affects the expression of a variety of genes, including proto-oncogenes and cancer suppressor genes. Changes in the activity of proto-oncogenes and cancer suppressor genes are crucial in tumorigenesis (4). Several DNA repair genes have been confirmed to play an important role in the development and prognosis of HNSCC (5–7). Hence, constructing a risk model composed of DNA repair genes may be useful for predicting the prognosis of patients with HNSCC.

In this study, we aimed to establish a prognostic prediction model for HNSCC based on DNA repair genes. We first equally divided all patients with HNSCC into training and testing sets. In the training set, we screened prognostic-related DNA repair genes using univariate proportional hazard (Cox) regression analysis and least absolute shrinkage and selection operator (LASSO) regression analysis to construct a risk model (8). All patients with HNSCC were classified into high- and low-risk groups according to the median value of the training set risk score. Subsequently, we verified the prognostic relevance and prognostic predictive ability of the risk model in the training and testing sets. We also analyzed the tumor-infiltrating immune cells, immune-related gene expression, tumor mutation burden, and drug sensitivity of patients with HNSCC in the high- and low-risk groups. The results showed that the risk model composed of DNA repair genes could effectively distinguish patients with different clinical outcomes and has independent predictive prognostic ability.

## Methods

### Data Download

The transcriptome profiling (RNA-seq) data harmonized to fragments per kilobase million (FPKM), clinical information, and tumor mutations in patients with HNSCC were downloaded from the Cancer Genome Atlas (TCGA) database (https://portal.gdc.cancer.gov/) in March 2021 (9). The pathologic stages were reconfirmed according to the seventh edition of the American Joint Committee on Cancer staging system (10). The gene transfer format (GTF) files were downloaded from Ensembl (http://asia.ensembl.org) for annotation (11). Immune-related genes were downloaded from the Tracking Tumor Immunophenotype (http://biocc.hrbmu.edu.cn/TIP/index.jsp) (12). The gene list, containing 569 DNA repair genes, was downloaded from Gene Set Enrichment Analysis (GSEA), “GO_DNA REPAIR” gene set (http://www.gsea-msigdb.org/gsea/msigdb/cards/GOBP_DNA_REPAIR.html) (13, 14). After annotation by the GTF files, 545 DNA repair genes were eventually used for subsequent analyses. GSE41613 (15), GSE27020 (16), GSE117973 (17), and GSE65858 (18) datasets with transcriptome and clinical data of patients with HNSCC were downloaded from Gene Expression Omnibus (https://www.ncbi.nlm.nih.gov/geo/) for external validation.

### Construction of Risk Model

To construct the risk model, we first combined the transcriptome data and clinical information of patients with HNSCC to obtain 499 samples with complete clinical information and transcriptome information, and then randomly divided them into a training set and a testing set on average. Subsequently, LASSO regression analysis was performed to further screen out 13 more representative DNA repair genes for use in constructing the risk model, and the correlation coefficients (*Coef*) and expression (*EXP*) of these 13 genes were obtained using the “glmnet” package in R (19). Finally, the risk score of each patient was calculated by the following formula: $\text{Risk Score}=\Sigma_{i=1}^{n}\;Expi\times Coefi$, where *n* refers to the number of selected DNA repair genes, *Expi* indicates the expression levels of gene *i* in each HNSCC sample, and *Coefi* is the correlation coefficient of gene *i*. Finally, we classified all HNSCC samples into high- and low-risk groups based on the median value of the risk score of the training set.

### Validation of the Risk Model

We verified the risk model separately in the training and testing sets. To this end, we first performed principal component analysis (PCA) in the training and testing sets to evaluate the discrimination of the risk model for patients in the high- and low-risk groups. We then utilized heat maps to show the expression patterns of the DNA repair genes in the risk model in the training and testing sets. The Kaplan-Meier survival curve was used to distinguish the difference in the clinical outcome of patients in the high- and low-risk groups, and the significant difference *P*-value was calculated by the log-rank test. The area under the curve (AUC) of the receiver operating characteristic (ROC) curve was used to evaluate the prognostic diagnostic accuracy of the risk score and clinical characteristics. Univariate and multivariate Cox regression analyses of risk score and clinical characteristics were used to evaluate the independent correlation between the risk score and prognosis of patients with HNSCC. We also performed the above verification in all patients with HNSCC. Then we divided all samples into multiple clinical subgroups based on clinical characteristics, and the Kaplan-Meier survival curve was performed in each subgroup to demonstrate the good prognostic ability of the risk score.

### Evaluation of the Tumor Immune Microenvironment and Immune-Related Gene Expression

Before analyzing the immune-infiltration situation using the CIBERSORT algorithm, which contains 22 types of immune cells, we first standardized the gene expression data through the “CIBERSORT” package in R (20). The Wilcoxon test was used to compare the different infiltrations of the 22 immune cells in the high- and low-risk groups. The Pearson test was used to analyze the correlation between risk genes and tumor-infiltrating immune cells through Statistical Product and Service Solutions 25.0 (SPSS 25.0) (21). The ESTIMATE (Estimation of STromal and Immune cells in MAlignant Tumors using Expression data) algorithm was used to evaluate the immune score, stromal cell content, and ESTIMATE score of each sample (22). We analyzed the expression of negative regulatory immune genes in the high- and low-risk groups using the Wilcoxon test. Finally, as the research on the role of immune checkpoint genes in various tumors is increasing, we analyzed the correlation between these genes and risk scores using the Spearman test and analyzed their differences in expression in the high- and low-risk patients using the Wilcoxon test.

### Assessment of Tumor Mutation Burden

We displayed the 30 genes with the highest mutation rate in all HNSCC samples and calculated the tumor mutation burden (TMB) of all samples through the “maftools” package in R (23). We then divided the HNSCC samples into high- and low-TMB groups according to the best cut-off value of the TMB of each sample. The Kaplan-Meier survival curve showed the clinical outcome of the two groups of patients with HNSCC. By combining the TMB groups and the risk groups, we further evaluated the impact of the risk score and tumor mutation burden on the clinical outcome of patients with HNSCC and displayed them with survival curves.

### Online Website Verification

We verified the influence of the expression of the 13 DNA repair genes on the Oncolnc website (http://www.oncolnc.org).

### Analysis of Drug Sensitivity

To evaluate the model in the clinical treatment of HNSCC, we calculated the half-inhibitory concentration (IC50) of chemotherapeutic drugs for HNSCC. The difference in the IC50 between the high- and low-risk groups was compared by Wilcoxon signed-rank test using the “pRRophetic” package in R (24).

### Statistical Analysis

The significance level of the *P*-value was set to <0.05. All statistical analyses were performed using R 4.0.4 (https://www.r-project.org/).

## Results

### Development and Validation of the Prognostic Model

**Figure 1:**
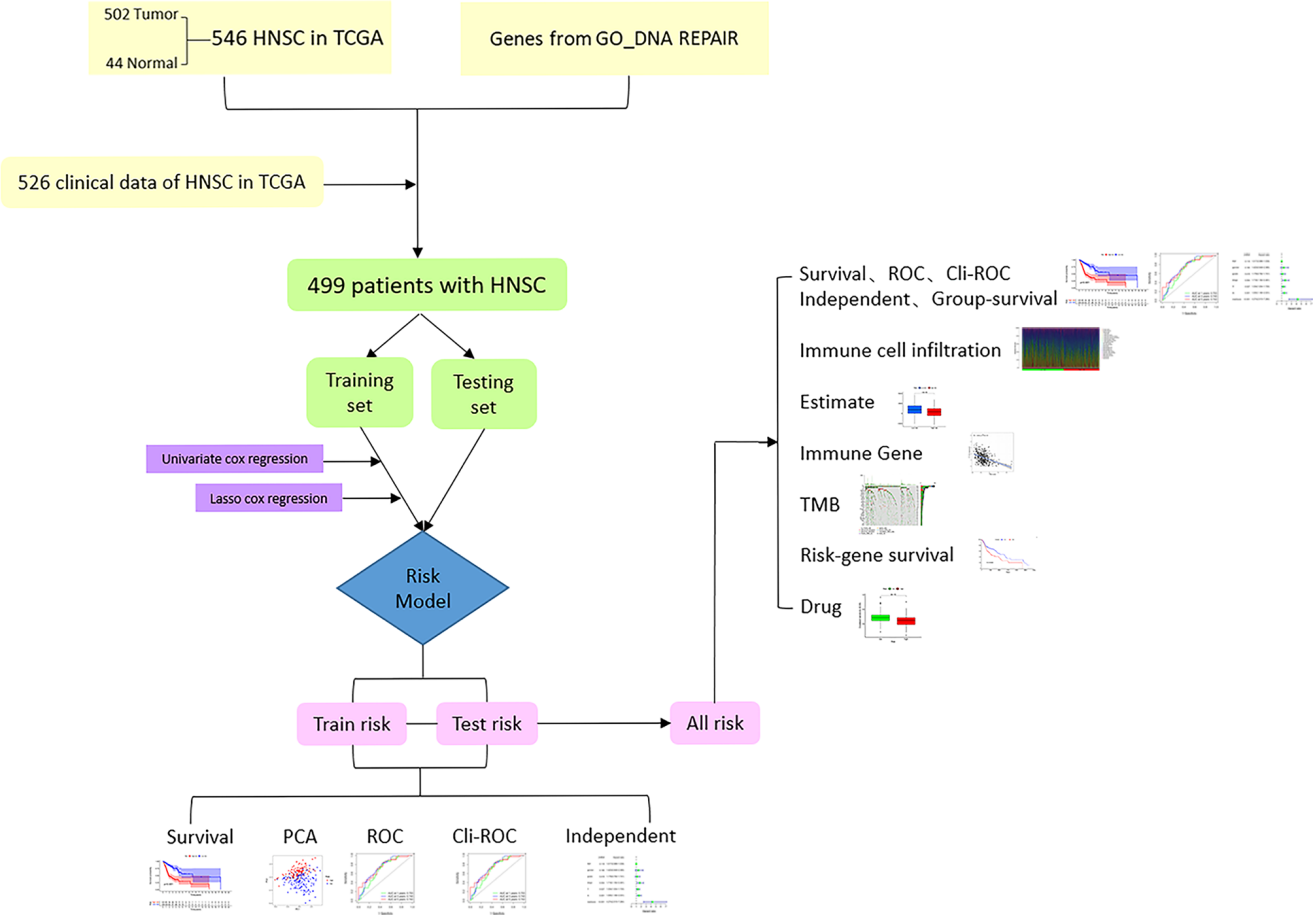
Flowchart of this study.

The flowchart of this research is shown in **Figure 1**. After merging the transcriptome data and the clinical data of patients with HNSCC downloaded from TCGA, we obtained 499 samples with complete information. We then divided all HNSCC samples into a training set (n = 251) and a testing set (n = 248). The basic clinical information of the two groups of patients is shown in **Table 1**. Subsequently, we screened out 82 prognostic-related genes among 545 DNA repair genes through univariate Cox regression analysis (**Table S1**, *P* < 0.05), of which 21 were risk genes (hazard ratio > 1). Subsequently, we screened out a further 13 representative DNA repair genes through LASSO Cox regression analysis, which were used to construct the risk model. The risk score was calculated based on the sum of the product of the expression (*Exp*) of all genes in the model and its correlation coefficient (*Coef*). The formula of the risk score was as follow: Risk Score = MORF4L2 * (0.0037) + COPS2 * (0.0063) + USP10 * (0.0255) + WAS * (–0.0123) + UVSSA * (–0.1324) + PRRX1 * (–0.0148) + ZBTB1 * (–0.0632) + DCLRE1C * (–0.0502) + MSH5 * (–0.3824) + DOT1L * (–0.1573) + ZBTB7A * (–0.00610) + POLR2C * (0.0085) + MORF4L1 * (0.0047). A negative correlation coefficient indicated that the gene was a protective factor in patients with HNSCC. In contrary, the gene with a positive correlation coefficient was a risk factor.

**Table 1:** Basic clinical information of training set and testing set.

Characteristics	Training set (n = 251)	Testing set (n = 248)
Age		
<=65	162	155
>65	89	93
Gender		
Female	70	63
Male	181	185
Stage		
Stage I-III	83	86
Stage IV	130	127
Unknown	36	35
Tumor		
T 1-2	85	87
T 3-4	136	134
Unknown	30	27
Lymph node		
N 0	84	83
N 1-3	122	118
Unknown	45	47

**Figure 2:**
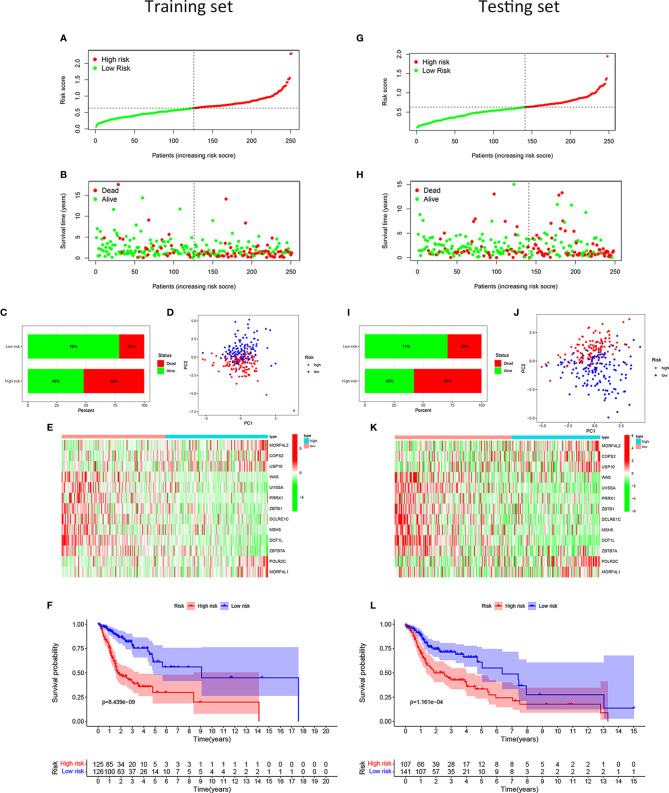
Development and validation of the risk model for patients with HNSCC. Distribution of the HNSCC samples with different risk scores in the training set **(A)**. According to the median value, the HNSCC samples were divided into high- (red dot) and low-risk (green dot) groups. The distribution of survival status of HNSCC samples **(B)**. The red dot indicated dead status, and the green dot indicated alive status. Percentage of patients with HNSCC in alive or dead status **(C)**. The red bar meant dead status, and the green bar meant alive status. PCA of HNSCC samples **(D)**. The red dots indicated HNSCC samples in the high-risk group, while the blue dot meant low risk. Heat map depicting the expression patterns in the 13 DRGs between high- and low-risk groups **(E)**. Kaplan-Meier survival curve demonstrating the clinical outcome differences between high- and low-risk groups **(F)**. In the testing set, the distribution of the risk scores among all HNSCC samples **(G)**. The distribution of survival status of HNSCC samples **(H)**. Percentage of patients in survival status and death status **(I)**. PCA of HNSCC samples **(J)**. Heat map depicting the expression differences in the 13 DRGs between high- and low-risk groups **(K)**. Kaplan-Meier survival curve showing the clinical outcome differences between the two groups **(L)**.

After calculating the risk scores of all patients with HNSCC, we divided the training set and testing set samples into high- and low-risk groups according to the median value of the training set risk score, as shown in **Figures 2A, G**. We found that in both the training and testing sets, the proportion of patients with HNSCC who died in the high-risk group was higher than that in the low-risk group (**Figures 2B, C, H, I**). The high- and low-risk groups were well distinguished (**Figures 2D, J**). Moreover, the DNA repair genes in the risk model showed the same expression pattern in the training and testing sets (**Figures 2E, K**). The Kaplan-Meier survival curve showed that the clinical outcomes of patients in the low-risk group were better than those in the high-risk group (**Figure 2L**), both in the training set (*P* = 8.439e–09; **Figure 2F**) and the testing set (*P* = 1.161e-04; **Figure 2L**).

**Figure 3:**
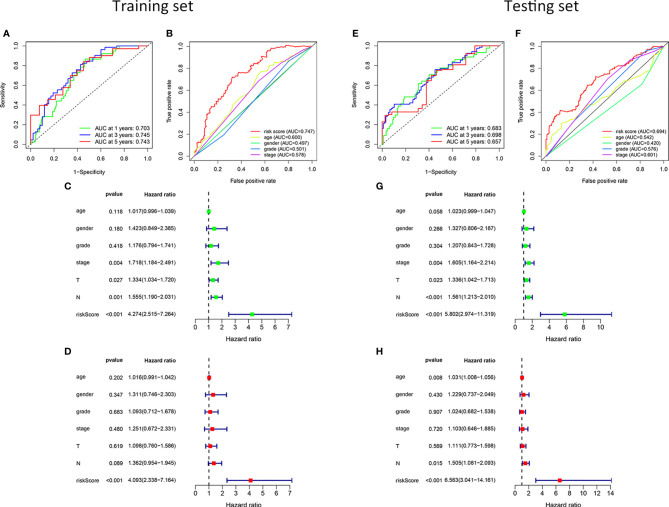
Validation of the risk model. In the training set, the 1-, 3-, and 5-year ROC curves **(A)**. The ROC curves of clinicopathological characteristics and risk score for 3-year OS **(B)**. In the testing set, the ROC curves for 1-, 3-, and 5-year OS **(E)**. The ROC curves of clinicopathological characteristics and risk score for 3-year OS **(F)**. Univariate and multivariate Cox regression survival analysis was used to validate whether age, gender, grade, stage, T, N, and risk score could independently predict the clinical outcome of patients with HNSCC in the training **(C, D)** and testing sets **(G, H)**.

To verify the ability and independence of our model to predict the prognosis of patients with HNSCC, we conducted ROC curves and univariate and multivariate Cox regression analyses in the training and testing sets, respectively. The sensitivity and specificity of the risk score were assessed using the ROC curve. In the training set, the area under the curve (AUC) of the 1-, 3-, and 5-year ROC curves of the risk score were all >0.7 (**Figure 3A**). The risk score had the largest AUCs of the 3-year ROC curve, compared to the clinical traits of patients with HNSCC (**Figure 3B**). In the testing set, the AUCs of the 1-, 3-, and 5-year ROC curves were all >0.65 (**Figure 3E**), and the risk score also had the largest AUCs of the 3-year ROC curve (**Figure 3F**). The hazard ratio (HR) value of the risk score was the largest in the univariate and multivariate Cox regression analyses of the risk score and multiple clinical features, which showed that the risk score was an independent prognostic factor (**Figures 3C, D**). The independence of the risk score for predicting the prognosis of HNSCC was confirmed in the test set (**Figures 3G, H**). **Table S2** shows the univariate and multivariate Cox regression analyses of the training and testing sets. Overall, the risk score was an independent prognostic factor for HNSCC. We were unable to find an external validation dataset with transcripts of all risk genes. However, we still verified the predictive ability of other genes except MSH5 in patients with HNSCC in GSE41613. We found that despite the lack of MSH, patients with HNSCC in the low-risk group showed better clinical outcomes than those in the high-risk group in our model (*P* < 0.05), and the expression pattern of the remaining genes was consistent with the training and testing sets (**Figure S1**). And we still verified in GSE117973 without transcript of UVSSA, GSE27020 without transcripts of MSH5 and UVSSA, and GSE65858 without transcripts of UVSSA and ZBTB1. The differences of prognosis of patients with HNSCC in the high- and low-risk groups were not significant (*P* > 0.05, **Figure S2**). For these external validation, we did a sensitivity analysis by using only 12 risk genes to recalculate the risk score. And we found that deleting every risk gene had little effect on the Kaplan-Meier survival curves (**Figures S3****,** **S4**).

**Figure 4:**
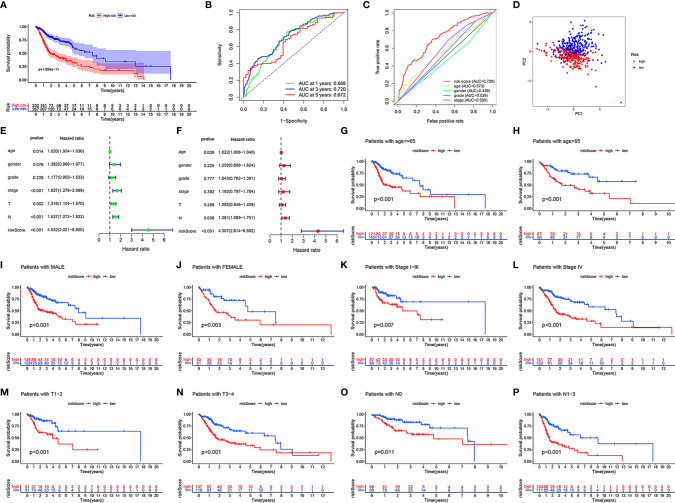
Validation in different clinical traits subgroups. In all HNSCC samples, the Kaplan-Meier survival curve demonstrating the clinical outcome differences between the high- and low-risk groups **(A)**. The ROC curves for 1-, 3-, and 5-year OS **(B)**, ROC curves of clinicopathological characteristics and risk score **(C)** for 3-year OS. PCA of all HNSCC samples **(D)**. Univariate and multivariate Cox regression survival analysis validated whether age, gender, grade, stage, T, N, and risk score could independently predict the clinical outcomes of patients with HNSCC **(E, F)**. Kaplan-Meier curves showing the differences in prognosis between the high- and low- risk groups in different clinical subgroups, including ≤65 **(G)**, >65 **(H)**, male **(I)**, female **(J)**, stage I-III **(K)**, stage IV **(L)**, T1-2 **(M)**, T3-4 **(N)**, N0 **(O)**, and N1-3 **(P)**.

To further verify the accuracy of the model, we divided all samples into clinical subgroups based on different clinical traits, and we analyzed differences in the clinical outcomes of high- and low-risk samples in each clinical subgroup. Before clinical subgroup validation, we conducted a risk model validation for all samples. The Kaplan-Meier survival curve of all patients showed that the clinical outcomes of patients in the low-risk group were significantly better than those in the high-risk group (*P* = 1.884e-11; **Figure 4A**). The sensitivity and specificity of the risk scores of all HNSCC samples were assessed using the ROC curve. The AUCs of the ROC curves of risk score for 1-, 3-, and 5-year were all >0.65 (**Figure 4B**). The risk score had the largest AUCs of the ROC curves for 3-year compared to the clinical traits of patients with HNSCC (**Figure 4C**). PCA showed that patients in the high- and low-risk groups showed good discrimination (**Figure 4D**). The HR value of the risk score was the highest in the univariate and multivariate regression analyses of risk score and clinical characteristics (**Figures 4E, F**). Details of the univariate and multivariate Cox regression analyses of the training and testing sets are shown in **Table S3**. We divided all HNSCC samples into different clinical subgroups according to age, gender, stage, tumor (T), and lymph node (N) of patients with HNSCC. The clinical outcomes of patients in the low-risk group were significantly better than those in the high-risk group in all clinical subgroups, including those aged ≤65 years (*P* < 0.001, **Figure 4G**) and >65 years (*P* < 0.001, **Figure 4H**), male (*P* < 0.001, **Figure 4I**) and female (*P* = 0.003, **Figure 4J**), stage I-III (P = 0.007, **Figure 4K**), stage IV (P < 0.001, **Figure 4L**), T1-2 (*P* < 0.001, **Figure 4M**) and T3-4 (*P* < 0.001, **Figure 4N**), and N0 (*P* = 0.011, **Figure 4O**), and N1-3 (*P* < 0.001, **Figure 4P**).

### Evaluation of the Immune Microenvironment and Expression of Immunoregulatory Genes

**Figure 5:**
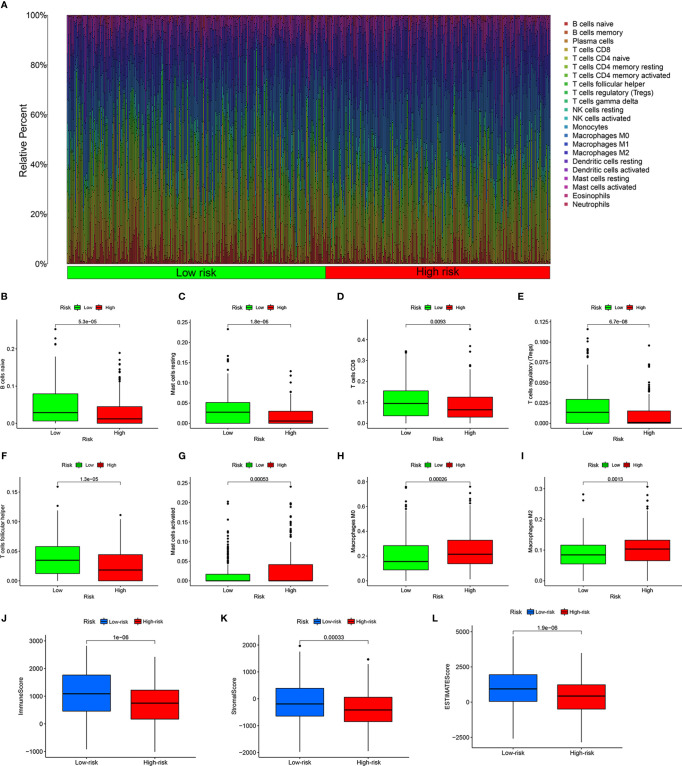
Estimation of the immune microenvironment. **(A)** Relative percentage of 22 types of tumor-infiltrating immune cells from the CIRBERSORT. Greater infiltration of B cells naïve **(B)**, resting mast cells **(C)**, CD8 T cells **(D)**, regulatory T cells **(E)**, and follicular helper T cells **(F)** in the low-risk group, and more infiltrating activated mast cells **(G)**, M0 macrophages **(H)**, and M2 macrophages **(I)** in the high-risk group. Higher immune score **(J)**, stromal score **(K)**, and ESTIMATE score **(L)** calculated by ESTIMATE in the low-risk group.

To reveal the differences in the immune microenvironment of high- and low-risk groups, including immune cell infiltration and expression of immunoregulatory and immune checkpoint genes, we first used the bioinformatics algorithm CIBERSORT to estimate 22 types of tumor-infiltrating immune cells in HNSCC. First, we found that among these 22 cell types, acquired immune-related immune cells infiltrated to a greater extent in HNSCC samples (**Figure 5A**). There were more naïve B cells (*P* = 5.3e-05, **Figure 5B**), resting mast cells (*P* = 1.8e-06, **Figure 5C**), T cells CD8 (*P* = 0.0093, **Figure 5D**), regulatory T cells (Tregs, *P* = 6.7e-08, **Figure 5E**), and follicular helper T cells (*P* = 1.3e-05, **Figure 5F**) in the low-risk group. In contrary, activated mast cells (*P* = 0.00053, **Figure 5G**), M0 macrophages (*P* = 0.00026, **Figure 5H**), and M2 macrophages (*P* = 0.0013, **Figure 5I**) showed greater infiltration in the high-risk group. The HNSCC samples in the low-risk group had higher immune scores (*P* = 1e-06, **Figure 5J**), stromal scores (*P* = 0.00033, **Figure 5K**), and ESTIMATE scores (*P* = 1.9e-06, **Figure 5L**) evaluated by ESTIMATE than the high-risk group. In other words, the tumor purity of HNSCC was lower in the low-risk group. Naïve B cells were positively correlated with eight risk genes that had negative correlation coefficients and negatively correlated with MORF4L2, which had a positive correlation coefficient (*P* < 0.05). CD8+ T cells were positively correlated with five risk genes that had negative correlation coefficients and negatively correlated with eight risk genes that had negative correlation coefficients (*P* < 0.05). Tregs and follicular helper T cells were positively correlated with all risk genes that had negative correlation coefficients and negatively correlated with all risk genes that had positive correlation coefficients (*P* < 0.05). Monocytes and Macrophages M2 were negatively related to most risk genes (*P* < 0.05). Macrophages M0 were negatively correlated with some risk genes that had negative correlation coefficients and positively correlated with some risk genes that had positive correlation coefficients (*P* < 0.05). Details are shown in **Table S4**.

**Figure 6:**
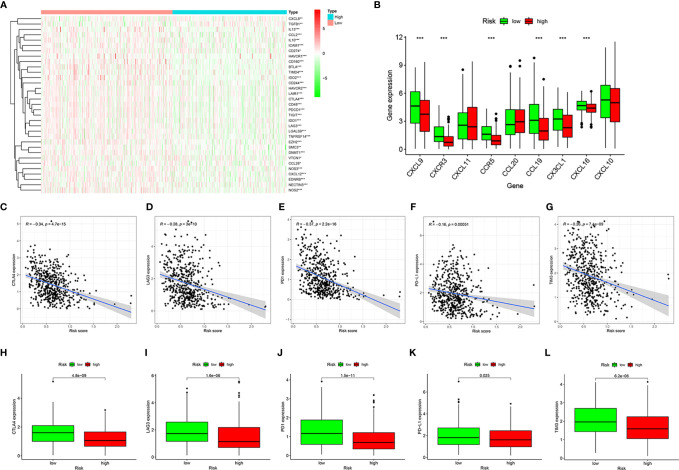
Estimation of immune regulatory gene expression. Heatmap of negative immune regulatory gene expression **(A)**. Differential expression of CD4+ T cell and CD8+ T cell regulatory genes in the high- and low-risk groups **(B)**. Correlation between gene expression and risk scores of CTLA4 **(C)**, LAG3 **(D)**, PD1 **(E)**, PD-L1 **(F)**, and TIM3 **(G)**. Differential expression of CTLA4 **(H)**, LAG3 **(I)**, PD1 **(J)**, PD-L1 **(K)**, and TIM3 **(L)** genes in high- and low-risk groups. *P < 0.05, **P < 0.01, ***P < 0.001.

Next, we analyzed the relevant immune regulatory genes to further reveal the differences in the immune microenvironment of HNSCC in the high- and low-risk groups. Almost all negative immune regulatory genes in **Figure 6A** were highly expressed in the low-risk group, similar to CD4+ T cell and CD8+ T cell regulatory genes (**Figure 6B**). In addition, in recent years, immune checkpoint inhibitors have become increasingly common in the treatment of various tumors, including HNSCC. Therefore we investigated whether the risk model was related to immune checkpoint inhibitor-related biomarkers by Spearman correlation analysis, and we discovered that high risk scores were negatively correlated with the expression of CTLA4 (*R* = −0.34, *P* = 4.7e-15, **Figure 6C**), LAG3 (*R* = −0.28, *P* = 3e-10, **Figure 6D**), PD1 (*R* = −0.37, *P* < 2.2e-16, **Figure 6E**), PD-L1 (*R* = −0.16, *P* = 0.00051, **Figure 6F**), and TIM3 (*R* = −0.26, *P* = 7.4e-09, **Figure 6G**). A further Wilcoxon rank test also confirmed the expression pattern of CTLA4 (*P* = 4.8e-09, **Figure 6H**), LAG3 (*P* = 1.6e-06, **Figure 6I**), PD1 (*P* = 1.5e-11, **Figure 6J**), PD-L1 (*P* = 0.025, **Figure 6K**), and TIM3 (*P* = 6.2e-06, **Figure 6L**).

### Assessment of Tumor Mutation Burden

**Figure 7:**
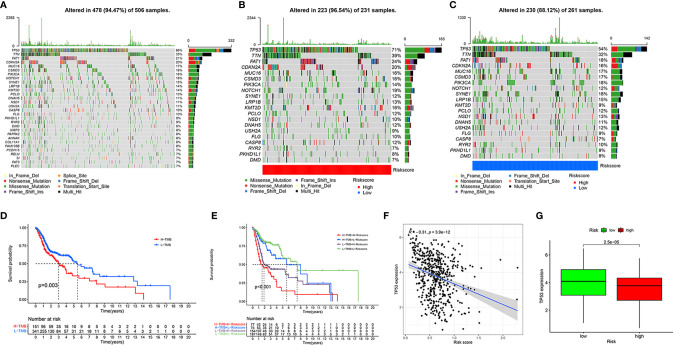
Assessment of tumor mutation burden of HNSCC. Top 30 mutant genes of all HNSCC samples **(A)**. Top 20 mutant genes of high- **(B)** and low-risk **(C)** groups. Kaplan-Meier survival curve showing the OS differences between the high- and low-TMB groups **(D)**. Kaplan-Meier survival curve showing the OS differences in the four combinations of TMB and risk **(E)**. Correlation of TP53 expression and risk score **(F)**. Different expression of TP53 in high- and low-risk groups **(G)**.

To determine the tumor mutation burden (TMB), we first downloaded all the mutation data of HNSCC from TCGA and showed the top 30 mutation rate genes (**Figure 7A**). Subsequently, we identified the genes with the top 20 mutation rates in the high- and low-risk groups (**Figures 7B, C**). The tumor mutation rate of high-risk group samples was slightly higher than that of patients in the low-risk group, and the gene with the highest mutation rate in the high- and low-risk groups samples was TP53. According to the best cut-off point of TMB, all patients with HNSCC were divided into high- and low-TMB groups. The Kaplan-Meier survival curve showed that the clinical outcomes of patients with low TMB were significantly better than those of patients with high TMB (*P* = 0.003, **Figure 7D**). To further evaluate the influence of TMB and risk score on the prognosis of patients with HNSCC, we combined the TMB group with the risk group and analyzed the clinical outcomes of different groups using the Kaplan-Meier survival curve. The results showed that patients with low risk and low TMB had the best clinical outcome, followed by patients with low risk and high tumor mutation load, and that patients with high risk and high tumor mutation load had the worst clinical outcome (*P* < 0.001, **Figure 7E**). Considering the high mutation rate of TP53, we analyzed the correlation between TP53 and the risk score and its expression in the high- and low-risk groups. As a result, we found that TP53 was negatively correlated with the risk score (*R* = −0.31, *P* = 3.9e-12, **Figure 7F**) and was highly expressed in the low-risk group (*P* = 2.5e-05, **Figure 7G**).

### Validation of the Website Oncolnc

**Figure 8:**
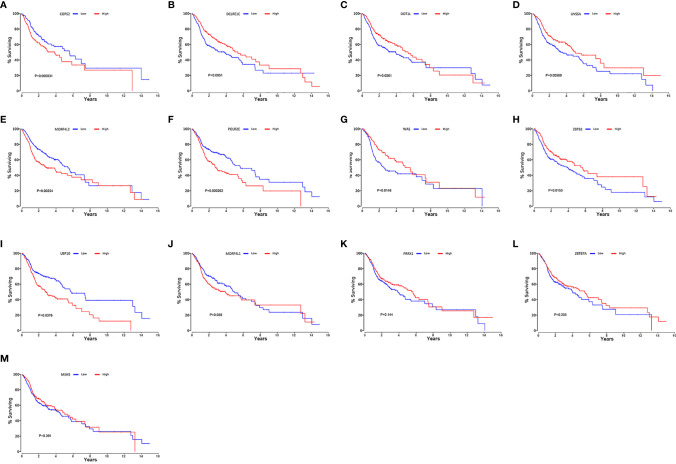
Verification of online website Oncolnc. Kaplan-Meier survival curve from Oncolnc (http://www.oncolnc.org/) of COPS2 **(A)**, DCLRE1C **(B)**, DOT1L **(C)**, UVSSA **(D)**, MORF4L2 **(E)**, POLR2C **(F)**, WAS **(G)**, ZBTB1 **(H)** and USP10 **(I)**, MORF4L1 **(J)**, PRRX1 **(K)**, ZBTB7A **(L)**, and MSH5 **(M)** for HNSCC.

We searched on the Oncolnc (http://www.oncolnc.org/) to verify the impact of high- and low-risk DRGs in the model on the prognosis of HNSCC and found that high-risk DRGs were correlated with poor prognosis and low-risk DRGs were associated with favorable patient prognosis. There were significant p-values for COPS2 (*P* = 0.000031, **Figure 8A**), DCLRE1C (*P* = 0.0051, **Figure 8B**), DOT1L (*P* = 0.0261, **Figure 8C**), UVSSA (*P* = 0.00589, **Figure 8D**), MORF4L2 (*P* = 0.00254, **Figure 8E**), POLR2C (*P* = 0.000262, **Figure 8F**), WAS (*P* = 0.0146, **Figure 8G**), ZBTB1 (*P* = 0.0153, **Figure 8H**), and USP10 (*P* = 0.0376, **Figure 8I**), whereas MORF4L1 (*P* = 0.088, **Figure 8J**), PRRX1 (*P* = 0.144, **Figure 8K**), ZBTB7A (*P* = 0.205, **Figure 8L**), and MSH5 (*P* = 0.391, **Figure 8M**) were not significant. The risk genes with negative correlation coefficients were also protective factors in the Oncolnc database.

### Analysis of Drug Sensitivity

**Figure 9:**
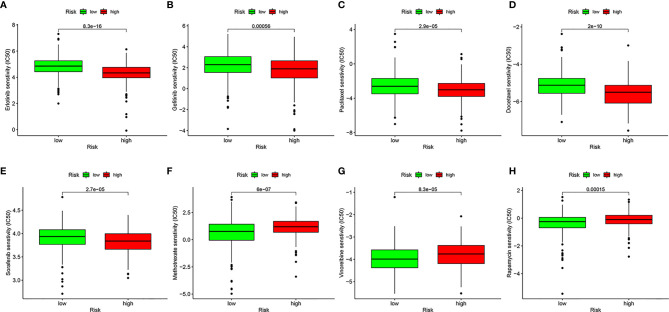
Analysis of drug sensitivity. Difference in inhibitory centration (IC50) of Erlotinib **(A)**, Gefitinib **(B)**, Paclitaxel **(C)**, Docetaxel **(D)**, Sorafenib **(E)**, Methotrexate **(F)**, Vinorelbine **(G)**, and Rapamycin **(H)** for treatment of HNSCC in the high- and low-risk groups.

To evaluate the possible clinical application of the risk model, we analyzed the sensitivity difference of chemotherapy drugs for HNSCC in the current stage of clinical trials between the high- and low-risk groups, with the drug sensitivity expressed by IC50. We showed that patients in the high-risk group were more sensitive to erlotinib (*P* = 8.3e-16, **Figure 9A**), gefitinib (*P* = 0.00056, **Figure 9B**), paclitaxel (*P* = 2.9e-05, **Figure 9C**), docetaxel (*P* = 2e-10, **Figure 9D**), and sorafenib (*P* = 2.7e-05, **Figure 9E**), whereas patients in low-risk group were more sensitive to methotrexate (*P* = 6e-07, **Figure 9F**), vinorelbine (*P* = 8.3e-05, **Figure 9G**), and rapamycin (*P* = 0.00015, **Figure 9H**), which indicated that the model could be used as a potential predictor of chemotherapy sensitivity.

## Discussion

An increasing number of studies have shown that DNA damage and repair play important roles in malignant tumors, including HNSCC (25). DNA repair has been proven to be widely involved in the development, prognosis, and metastasis of HNSCC (26). Further studies on the expression profile of DNA repair genes in HNSCC specimens may provide new ideas to improve the clinical prognosis of patients.

A total of 545 DNA repair genes were obtained from the “GO_DNA REPAIR” gene set of the GSEA database for subsequent analysis. Through univariate and LASSO Cox regression analyses in the training set, we constructed a risk model that included 13 DNA repair genes. Patients in high-risk group had worse clinical outcomes than low-risk patients. The AUC of the ROC at 1-, 3-, and 5-year confirmed the good prediction performance of the risk score. In addition, prediction accuracy and independence were verified using univariate and multivariate Cox regression analyses. We also performed clinical subgroup validation in the internal dataset and further validated the model in the online database Oncolnc, which reflected good accuracy and repeatability of the risk model.

We illustrated the immune landscape of patients with HNSCC using CIBERSORT and ESTIMATE, including tumor-infiltrating immune cells, immune score, immune regulatory genes, and immune checkpoint genes, all of which are considered important in HNSCC (27). Comprehensive analysis revealed that the risk score was more negatively related to tumor-infiltrating cells such as naïve B cells, resting mast cells, CD8+ T cells, Tregs, and follicular helper T cells, and positively related to activated mast cells and macrophages. According to **Table S4**, the correlation between risk score and tumor-infiltrating immune cells was contributed by the influence of all risk genes on tumor-infiltrating immune cells. Tumor-infiltrating immune cells both correlated with eight gene transcripts that have a negative correlation coefficient and five gene transcripts having a positive correlation coefficient. In addition, patients in the low-risk group had higher immune scores, stromal scores, and ESTIMATE scores, which indicated that their tumor purity was lower.

In this study, some of the DRGs in the risk model have already been identified as having an important role in the immune system while others have not been well studied in the immune system at present. Decreasing the activity of DOT1L (DOT1 like histone lysine methyltransferase) through silencing or an inhibitor preferentially suppressed the production of interleukin 6 (IL-6) and interferon β (IFN-β) but not of tumor necrosis factor α (TNF-α) in macrophages triggered by Toll-like receptor (TLR) ligands or virus infection. DOT1L-mediated selective histone 3 lysine 79 (H3K79me2/3) modifications at the IL-6 and IFN-β1 promoters are required for the full activation of innate immune responses (28). DO1L plays an important role in regulating the differentiation and complete function of CD4+, CD8+T cells and B cells in the process of acquired immunity, while DO1L knockdown or mutation invalidates acquired immunity (29–32). ZBTB1 (zinc finger and BTB domain containing 1) prevents DNA damage in replicating immune progenitors, allowing the generation of B cells, T cells, and myeloid cells (33). In alveolar macrophages, antigen presentation was ZBTB7A (zinc finger and BTB domain containing 7A)-dependent where alveolar macrophages deficient in ZBTB7A failed to induce antibody production and T cell responses (34).

CD8+ T cell infiltration indicates better prognosis of patients with HNSCC (35). Because of the negative correlation between the risk score and tumor-infiltrating cells, we investigated the differential expression of negative immune regulatory genes, CD4+ T cell and CD8+ T cell regulatory genes in different groups. The results showed that almost all of these genes were highly expressed in the low-risk group, potentially due to increased infiltration of immune cells in the low-risk group samples. Subsequently, the correlation between the risk score and the expression of five immune checkpoint genes, CTLA4, LAG3, PD1, PD-L1, and TIM3, indicated that the expression of immune checkpoint genes was negatively correlated with the risk score and was highly expressed in the low-risk group, suggesting that immune checkpoint inhibitors may be beneficial to patients with HNSCC with low risk scores.

In recent years, there has been an increasing number of studies on the TMB of various tumors, including HNSCC, not only in the context of its use as a biomarker, but also in the treatment of immune checkpoint inhibitors (36). In our study, TMB was positively correlated with risk score and poorer clinical outcomes. Because TP53 showed the highest mutation rate, we compared its expression in different groups and found that it was negatively correlated with the risk score and highly expressed in the low-risk group. Our model suggested that patients with HNSCC with high risk scores were more sensitive to biological inhibitors such as erlotinib, gefitinib, and sorafenib, instead of chemotherapeutics like methotrexate. These analyses of drug sensitivity were based on “pRRophetic” package in R (20). Although the authenticity of the difference in drug sensitivity of these drugs among patients with HNSCC in different risk groups needs to be verified by further clinical trials, this model based on DNA repair genes provides the possibility for guiding clinical drug use. We speculated that the effect of immunotherapy on HNSCC would be better than that of traditional chemotherapy.

In this study, some of the DRGs in the process of modeling that have already been identified play an important role in the malignant phenotypes of various cancer types. DOT1L is involved in tumorigenesis and tumor metabolism or metastasis of ovarian cancer (37, 38), prostate cancer (39, 40), leukemia (41, 42), neuroblastoma (43), colorectal cancer (44), and breast cancer (45). PRRX1 (paired related homeobox 1), a homeodomain transcriptional factor, has been demonstrated to be important in pancreatic cancer, especially in the regulation of epithelial-to-mesenchymal transition (EMT) in pancreatic cancer (46–49). Moreover, UPS10 (ubiquitin-specific peptidase 10), a deubiquitinase, promotes proliferation of hepatocellular carcinoma by deubiquitinating and stabilizing YAP/TAZ, and suppresses lung tumorigenesis by deubiquitinating and stabilizing KLF4 (50, 51). ZBTB7A (zinc finger and BTB domain containing 7A) acts as a tumor suppressor through transcriptional repression in several carcinomas (52–54). Moreover, its mutation or downregulation promotes cancer progression (55, 56). Furthermore, its homologous gene, ZBTB1, participates in regulating the treatment effectiveness and resistance to chemotherapy (57, 58). At present, other DRGs in the model have not been studied in depth in tumors.

In general, the prognosis model constructed based on the DNA repair gene transcripts and clinical information of patients with HNSCC in TCGA can well predict the prognosis of patients with HNSCC in the high- and low-risk groups. And this model systematically elaborated the molecular characteristics and immune microenvironment of HNSCC. The internal verification established based on the TCGA database also proved the stability of the model and provided reference value for prediction of the clinical outcomes of patients with HNSCC. In addition, the significant differences of multiple immune checkpoint genes between the high- and low-risk groups point out possible directions for the immunotherapy of patients with HNSCC.

However, we recognized that there were limitations to this study. On the one hand, the HNSCC samples involved in this study were not sufficient, and the DNA repair gene transcripts and clinical information of multiple GEO databases were incomplete, which hindered our external verification. On the other hand, the immaturity of the biobank of our institution was not enough to verify. Nevertheless, we still successfully completed external verification with the remaining genes in GSE41613 without MSH5 transcript, which further confirmed the availability and stability of the prognostic model. However, there were no significant differences in the Kaplan-Meier survival curves validated in the GSE117973 (without UVSSA), GSE27020 (without UVSSA and MSH5), and GSE65858 (without ZBTB1 and UVSSA). We assumed that the lack of a relatively important gene would reduce the predictive ability of the model, which might be the reason for the failure of the verification in GSE27020, GSE117973, and GSE65858.

## Conclusion

In conclusion, this study constructed a 13-DRG signature for the prognosis of HNSCC, which could accurately and independently predict the clinical outcome of the patient. We then revealed the immune landscape, TMB, and sensitivity to chemotherapy drugs in different risk groups, which might be used to guide clinical treatment decisions.

## Data Availability Statement

Publicly available datasets were analyzed in this study. This data can be found here: https://portal.gdc.cancer.gov/

http://asia.ensembl.org
http://biocc.hrbmu.edu.cn/TIP/index.jsp http://www.gsea-msigdb.org/gsea/msigdb/cards/GOBP_DNA_REPAIR.html
http://www.oncolnc.org/
https://www.r-project.org/
https://www.ncbi.nlm.nih.gov/geo/.

## Author Contributions

RM and EW designed the study, performed the experiments and plotted the data. RM and JW validated the data. RM and EW drafted the manuscript. HX, SZ, and JS reviewed and edited the manuscript. HX and SZ supervised the project. HX and SZ funded the experiments for the study. All authors contributed to the article and approved the submitted version.

## Funding

This study was supported by grants from the National Natural Science Foundation of China (Grant numbers 81771002, 82071057).

## Conflict of Interest

The authors declare that the research was conducted in the absence of any commercial or financial relationships that could be construed as a potential conflict of interest.

## Publisher’s Note

All claims expressed in this article are solely those of the authors and do not necessarily represent those of their affiliated organizations, or those of the publisher, the editors and the reviewers. Any product that may be evaluated in this article, or claim that may be made by its manufacturer, is not guaranteed or endorsed by the publisher.
