# Supplementary material for: The Prognostic Value of the DNA Repair Gene Signature in Head and Neck Squamous Cell Carcinoma
Source: Front Oncol. 2021 Jul 30;11:710694. doi: 10.3389/fonc.2021.710694 (PMC8362833; doi:10.3389/fonc.2021.710694)
Supplement: Supplementary file 1 [file DataSheet_1.docx]

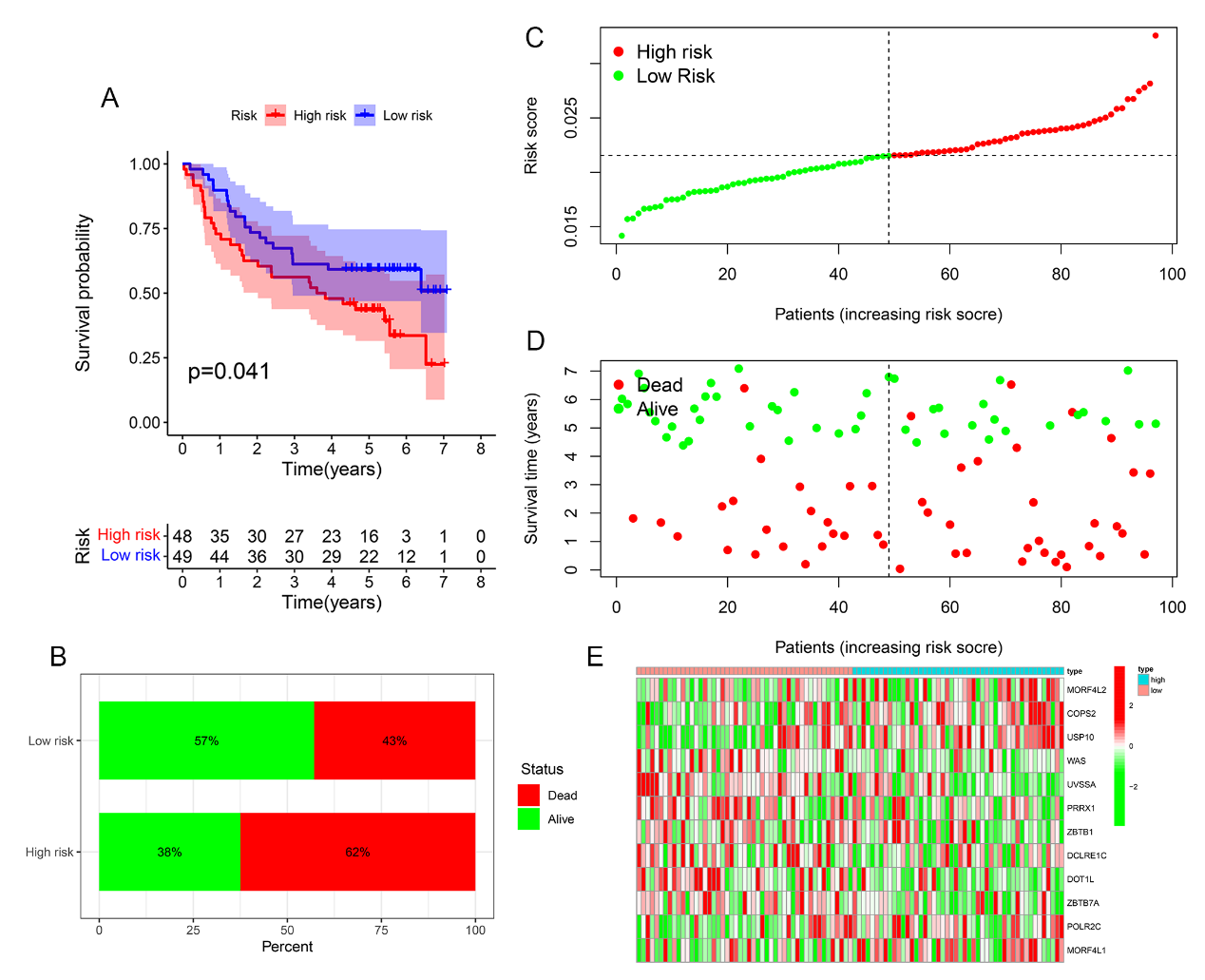


Figure S1. Verification in GSE41613. Kaplan-Meier survival curves of patients with HNSCC in high- and low-risk groups (A). Percentage of patients with HNSCC in alive or death status (B). The red bar meant dead status and the green bar meant alive status. Distribution of the HNSCC samples with different risk scores (C). According to the median value, the HNSCC samples were divided into high- (red dot) and low-risk (green dot) groups. The distribution of survival status of HNSCC samples (D). The red dot indicated dead and the green dot indicated alive. Heat map depicting the expression patterns in the 13 DRGs between high- and low-risk groups (E).
