# Supplementary material for: The Prognostic Value of the DNA Repair Gene Signature in Head and Neck Squamous Cell Carcinoma
Source: Front Oncol. 2021 Jul 30;11:710694. doi: 10.3389/fonc.2021.710694 (PMC8362833; doi:10.3389/fonc.2021.710694)
Supplement: Supplementary file 2 [file DataSheet_2.docx]

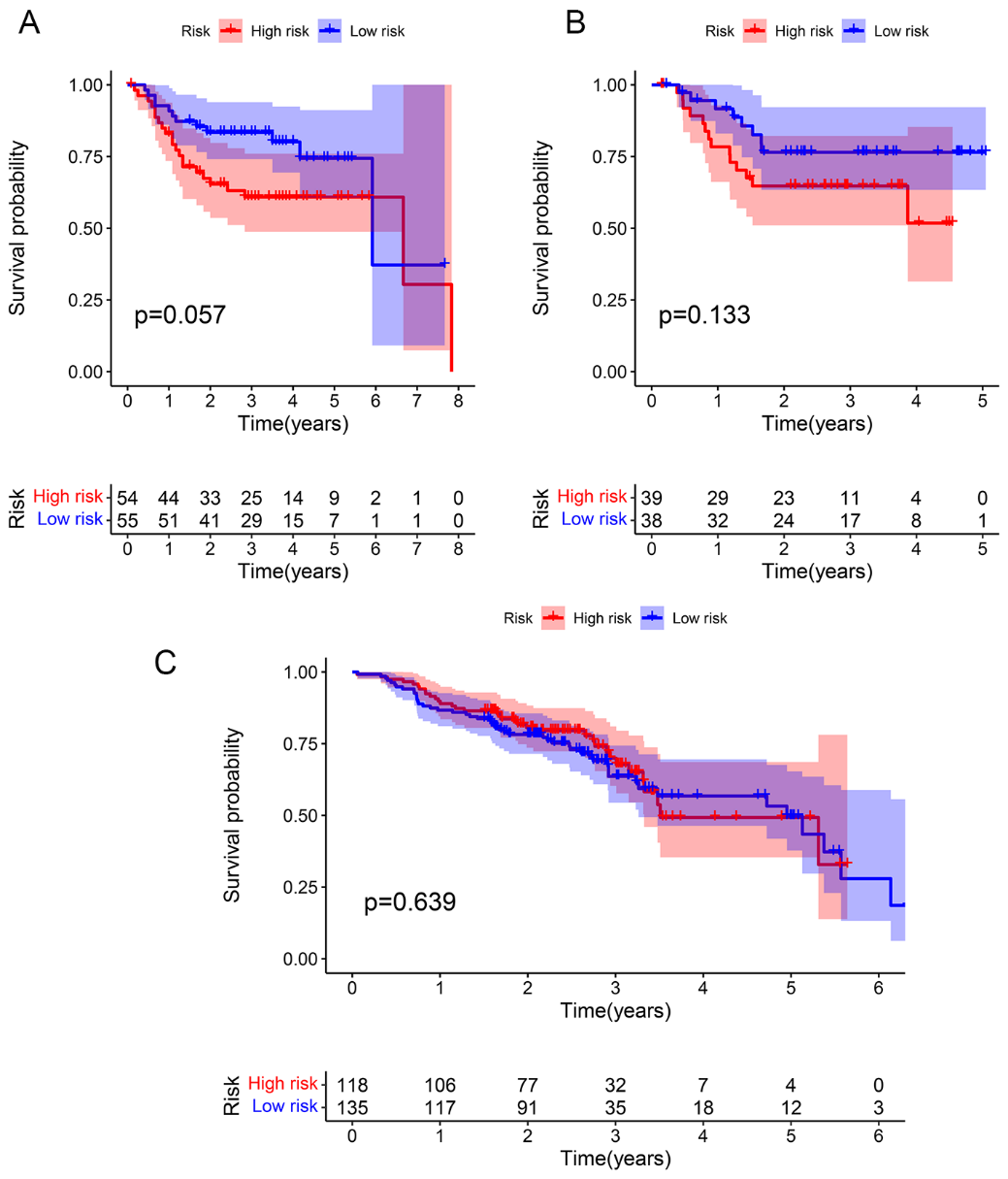


Figure S2. Verification in GSE27020, GSE117973, and GSE65858. Kaplan-Meier survival curves of disease free survival of patients with HNSCC in high- and low-risk groups in GSE27020 (A). Kaplan-Meier survival curves of progress free survival of patients with HNSCC in high- and low-risk groups in GSE117973 (B). Kaplan-Meier survival curves of overall survival of patients with HNSCC in high- and low-risk groups in GSE65858 (C).
