# Supplementary material for: The Prognostic Value of the DNA Repair Gene Signature in Head and Neck Squamous Cell Carcinoma
Source: Front Oncol. 2021 Jul 30;11:710694. doi: 10.3389/fonc.2021.710694 (PMC8362833; doi:10.3389/fonc.2021.710694)
Supplement: Supplementary file 4 [file DataSheet_4.docx]

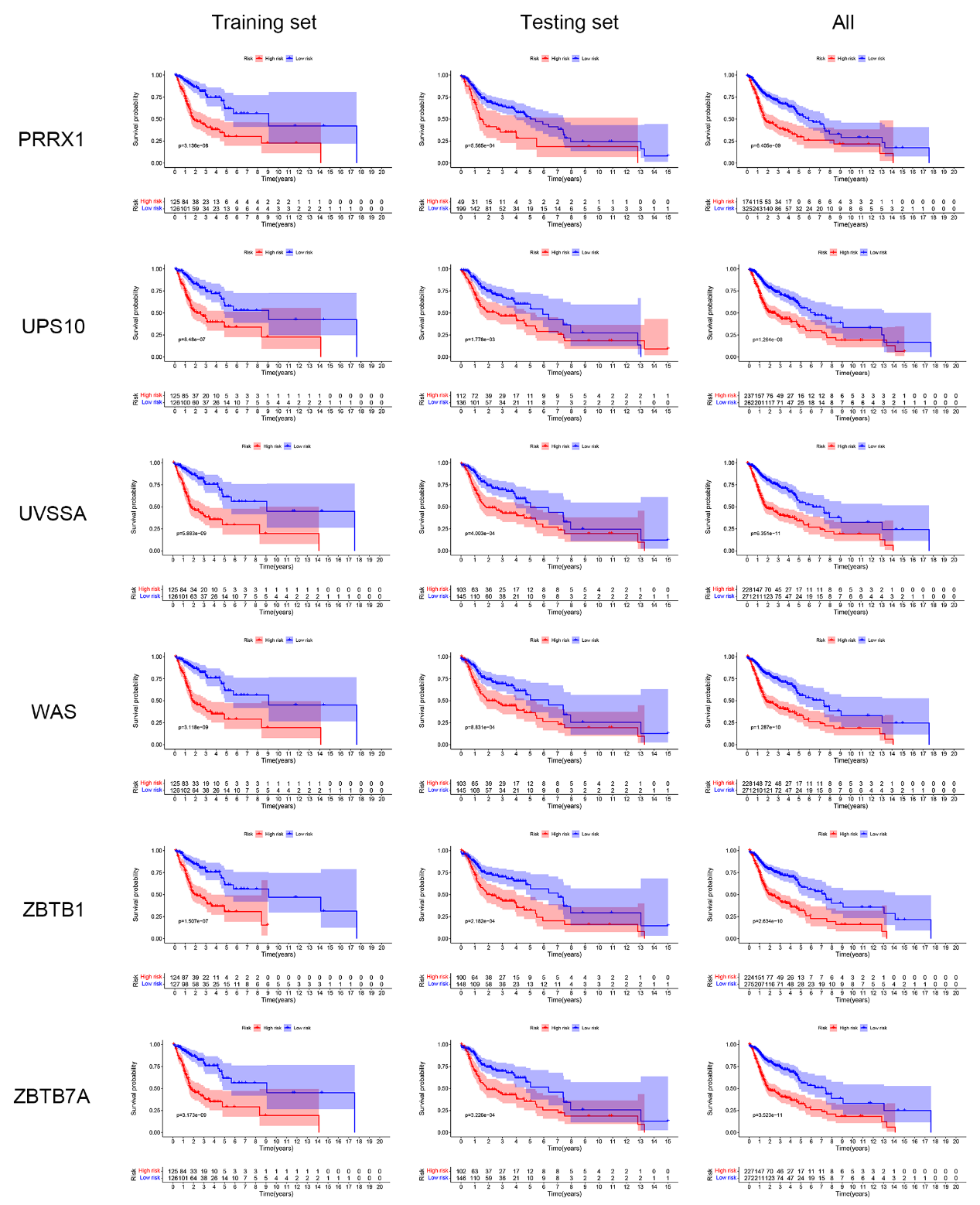


Figure S4. Sensitivity analysis of 6 risk genes. We deleted the risk genes one by one and recalculated the risk score. According to the median risk score of the training set, all patients were divided into high- and low-risk groups. Kaplan-Meier survival curves of patients in high- and low-risk groups in the risk model lacking PRRX1, UPS10, UVSSA, WAS, ZBTB1, and ZBTB7A.
