# Supplementary material for: The Prognostic Value of the DNA Repair Gene Signature in Head and Neck Squamous Cell Carcinoma
Source: Front Oncol. 2021 Jul 30;11:710694. doi: 10.3389/fonc.2021.710694 (PMC8362833; doi:10.3389/fonc.2021.710694)
Supplement: Supplementary file 5 [file Table_1.docx]

Table S1. Eighty-two prognostic-related DNA repair genes.

| Id | HR | HR.95L | HR.95H | p-value |
| --- | --- | --- | --- | --- |
| MORF4L2 | 1.011430 | 1.006115 | 1.016772 | 0.000024 |
| TENT4A | 0.957489 | 0.916804 | 0.999979 | 0.049889 |
| RAD9A | 0.930685 | 0.874617 | 0.990348 | 0.023456 |
| PAGR1 | 0.890475 | 0.815051 | 0.972879 | 0.010203 |
| ANKLE1 | 0.722555 | 0.549976 | 0.949288 | 0.019611 |
| RPA3 | 1.036459 | 1.007287 | 1.066475 | 0.013955 |
| POLH | 0.894682 | 0.828614 | 0.966018 | 0.004465 |
| CDC7 | 0.929848 | 0.869014 | 0.994940 | 0.035126 |
| USP1 | 0.973974 | 0.952049 | 0.996403 | 0.023198 |
| COPS2 | 1.076927 | 1.028032 | 1.128147 | 0.001771 |
| USP10 | 1.033359 | 1.007935 | 1.059424 | 0.009827 |
| WAS | 0.938100 | 0.898087 | 0.979896 | 0.004064 |
| FANCE | 0.974560 | 0.950768 | 0.998948 | 0.041009 |
| ATRIP | 0.000873 | 0.000002 | 0.463285 | 0.027784 |
| MARF1 | 0.895565 | 0.833168 | 0.962636 | 0.002759 |
| CDKN2D | 0.966739 | 0.937391 | 0.997007 | 0.031511 |
| ARID2 | 0.886560 | 0.796320 | 0.987026 | 0.027920 |
| TIMELESS | 0.976350 | 0.955028 | 0.998148 | 0.033625 |
| UVSSA | 0.578521 | 0.417573 | 0.801506 | 0.001001 |
| SUMO1 | 1.010489 | 1.001889 | 1.019163 | 0.016728 |
| MSH4 | 0.000555 | 0.000001 | 0.399116 | 0.025506 |
| JMY | 0.877972 | 0.795390 | 0.969128 | 0.009819 |
| IFFO1 | 0.764631 | 0.64842 | 0.90167 | 0.00142 |
| UBE2N | 1.031173 | 1.001774 | 1.061436 | 0.037521 |
| DMAP1 | 0.937365 | 0.879262 | 0.999308 | 0.047573 |
| MNAT1 | 1.052939 | 1.000814 | 1.10778 | 0.04644 |
| ESCO2 | 0.821392 | 0.693324 | 0.973116 | 0.022901 |
| NSD2 | 0.940751 | 0.888403 | 0.996183 | 0.036539 |
| DNA2 | 0.847971 | 0.72632 | 0.989997 | 0.03687 |
| PRRX1 | 0.978393 | 0.962528 | 0.994519 | 0.008822 |
| MCM3 | 0.99206 | 0.984825 | 0.999347 | 0.032778 |
| OTUB1 | 1.016853 | 1.002997 | 1.030901 | 0.016966 |
| SLX4 | 0.748674 | 0.587127 | 0.95467 | 0.019594 |
| FZR1 | 0.958078 | 0.926312 | 0.990934 | 0.012798 |
| ZBTB1 | 0.903368 | 0.840845 | 0.97054 | 0.005484 |
| WDR48 | 0.893753 | 0.820508 | 0.973536 | 0.010031 |
| CHD1L | 1.035297 | 1.002701 | 1.068953 | 0.033571 |
| UBE2A | 1.029332 | 1.00261 | 1.056765 | 0.031226 |
| POLE2 | 0.915628 | 0.840867 | 0.997037 | 0.042535 |
| RPA2 | 0.986576 | 0.975398 | 0.997882 | 0.020091 |
| NPLOC4 | 0.973746 | 0.94964 | 0.998465 | 0.037516 |
| LIG1 | 0.962487 | 0.932797 | 0.993122 | 0.016772 |
| GEN1 | 0.830058 | 0.71077 | 0.969367 | 0.018622 |
| POLD1 | 0.966286 | 0.938225 | 0.995186 | 0.022555 |
| DCLRE1C | 0.679484 | 0.538554 | 0.857292 | 0.001121 |
| PSMD14 | 1.044044 | 1.011518 | 1.077615 | 0.007604 |
| SLF2 | 0.89053 | 0.794117 | 0.998649 | 0.047357 |
| EYA1 | 1.144737 | 1.001975 | 1.307841 | 0.046702 |
| CHAF1A | 0.967207 | 0.942466 | 0.992597 | 0.011668 |
| PIAS4 | 0.897555 | 0.841604 | 0.957225 | 0.000998 |
| RTEL1 | 0.173287 | 0.035146 | 0.854402 | 0.031298 |
| BARD1 | 0.854297 | 0.763966 | 0.955309 | 0.005748 |
| EME2 | 0.78993 | 0.645655 | 0.966444 | 0.021925 |
| XAB2 | 0.965294 | 0.934264 | 0.997354 | 0.034098 |
| GINS4 | 0.885765 | 0.807418 | 0.971713 | 0.010251 |
| POLQ | 0.867164 | 0.76427 | 0.983911 | 0.026991 |
| TOPBP1 | 0.972874 | 0.950049 | 0.996246 | 0.023178 |
| TP73 | 0.94827 | 0.909441 | 0.988757 | 0.012774 |
| MSH5 | 0.267476 | 0.103273 | 0.692759 | 0.006608 |
| POLD2 | 1.00984 | 1.002354 | 1.017381 | 0.009896 |
| TONSL | 0.945301 | 0.897229 | 0.995948 | 0.03465 |
| EMSY | 0.809494 | 0.684308 | 0.957582 | 0.013677 |
| CETN2 | 1.01668 | 1.003353 | 1.030185 | 0.014005 |
| CDC14B | 0.797603 | 0.666559 | 0.954411 | 0.01353 |
| EXO5 | 0.832678 | 0.726009 | 0.95502 | 0.008845 |
| DOT1L | 0.823785 | 0.751968 | 0.902461 | 3.11E-05 |
| INO80B | 0.896503 | 0.805172 | 0.998195 | 0.046271 |
| POLE | 0.881362 | 0.804036 | 0.966123 | 0.007026 |
| EYA3 | 0.917205 | 0.850652 | 0.988965 | 0.024533 |
| ZBTB7A | 0.938155 | 0.899819 | 0.978123 | 0.002708 |
| INO80D | 0.739109 | 0.573972 | 0.951756 | 0.019118 |
| XRCC6 | 1.00293 | 1.000358 | 1.005508 | 0.02551 |
| DDX11 | 0.900488 | 0.835035 | 0.971071 | 0.006481 |
| POLR2C | 1.040561 | 1.017034 | 1.064633 | 0.000656 |
| RAD9B | 0.516071 | 0.299671 | 0.888739 | 0.017066 |
| NPM1 | 1.004266 | 1.002092 | 1.006444 | 0.000118 |
| COPS6 | 1.011003 | 1.003366 | 1.018699 | 0.004676 |
| BABAM2 | 1.053333 | 1.003509 | 1.105631 | 0.035586 |
| NSMCE3 | 1.0316 | 1.000355 | 1.06382 | 0.047414 |
| MORF4L1 | 1.019735 | 1.005291 | 1.034386 | 0.007252 |
| SMCHD1 | 0.933666 | 0.880854 | 0.989645 | 0.02087 |
| BRIP1 | 0.842622 | 0.736878 | 0.963541 | 0.012321 |
