# Supplementary material for: The Prognostic Value of the DNA Repair Gene Signature in Head and Neck Squamous Cell Carcinoma
Source: Front Oncol. 2021 Jul 30;11:710694. doi: 10.3389/fonc.2021.710694 (PMC8362833; doi:10.3389/fonc.2021.710694)
Supplement: Supplementary file 6 [file Table_2.docx]

Table S2. Cox hazard ratio analysis of clinical features and risk score in training and testing sets.

|  | Training set | | | | | |
| --- | --- | --- | --- | --- | --- | --- |
| Clinical features | Univariate | | | Multivariate | | |
|  | HR | 95%CI | *P*-Value | HR | 95%CI | *P*-Value |
| Age | 1.017 | 0.996-1.039 | 0.118 | 1.016 | 0.991-1.042 | 0.202 |
| Gender | 1.423 | 0.849-2.385 | 0.180 | 1.311 | 0.746-2.303 | 0.347 |
| Grade | 1.176 | 0.794-1.741 | 0.418 | 1.093 | 0.712-1.678 | 0.683 |
| Stage | 1.718 | 1.184-2.491 | 0.004 | 1.251 | 0.672-2.331 | 0.480 |
| T | 1.334 | 1.034-1.720 | 0.027 | 1.098 | 0.760-1.586 | 0.619 |
| N | 1.555 | 1.190-2.031 | 0.001 | 1.362 | 0.954-1.945 | 0.089 |
| Risk score | 4.274 | 2.515-7.264 | 0.000 | 4.093 | 2.338-7.164 | 0.000 |
|  | Testing set | | | | | |
| Clinical features | Univariate | | | Multivariate | | |
|  | HR | 95%CI | *P*-Value | HR | 95%CI | *P*-Value |
| Age | 1.023 | 0.999-1.047 | 0.058 | 1.032 | 1.008-1.056 | 0.008 |
| Gender | 1.327 | 0.806-2.187 | 0.266 | 1.229 | 0.737-2.049 | 0.430 |
| Grade | 1.207 | 0.843-1.728 | 0.304 | 1.024 | 0.682-1.538 | 0.908 |
| Stage | 1.605 | 1.164-2.214 | 0.004 | 1.103 | 0.646-1.885 | 0.720 |
| T | 1.336 | 1.042-1.713 | 0.023 | 1.111 | 0.773-1.598 | 0.569 |
| N | 1.561 | 1.213-2.011 | 0.001 | 1.505 | 1.081-2.093 | 0.015 |
| Risk score | 5.802 | 2.974-11.32 | 0.000 | 6.563 | 3.041-14.16 | 0.000 |
