# Supplementary material for: The Prognostic Value of the DNA Repair Gene Signature in Head and Neck Squamous Cell Carcinoma
Source: Front Oncol. 2021 Jul 30;11:710694. doi: 10.3389/fonc.2021.710694 (PMC8362833; doi:10.3389/fonc.2021.710694)
Supplement: Supplementary file 7 [file Table_3.docx]

Table S3. Cox hazard ratio analysis of clinical features and risk score in all HNSCC samples.

| Clinical features | Univariate | | | Multivariate | | |
| --- | --- | --- | --- | --- | --- | --- |
|  | HR | 95%CI | *P*-Value | HR | 95%CI | *P*-Value |
| Age | 1.020 | 1.004-1.036 | 0.014 | 1.022 | 1.006-1.040 | 0.009 |
| Gender | 1.382 | 0.966-1.977 | 0.076 | 1.258 | 0.868-1.824 | 0.225 |
| Grade | 1.177 | 0.903-1.533 | 0.228 | 1.043 | 0.782-1.391 | 0.777 |
| Stage | 1.627 | 1.279-2.069 | 0.000 | 1.192 | 0.797-1.784 | 0.392 |
| T | 1.316 | 1.104-1.570 | 0.002 | 1.092 | 0.846-1.409 | 0.498 |
| N | 1.527 | 1.273-1.832 | 0.000 | 1.381 | 1.089-1.751 | 0.008 |
| Risk score | 4.532 | 3.021-6.800 | 0.000 | 4.307 | 2.814-6.592 | 0.000 |
